# Supplementary figures and images for: Transforming Estonian health data to the Observational Medical Outcomes Partnership (OMOP) Common Data Model: lessons learned
Source: JAMIA Open. 2023 Dec 5;6(4):ooad100. doi: 10.1093/jamiaopen/ooad100 (PMC10697784; doi:10.1093/jamiaopen/ooad100)

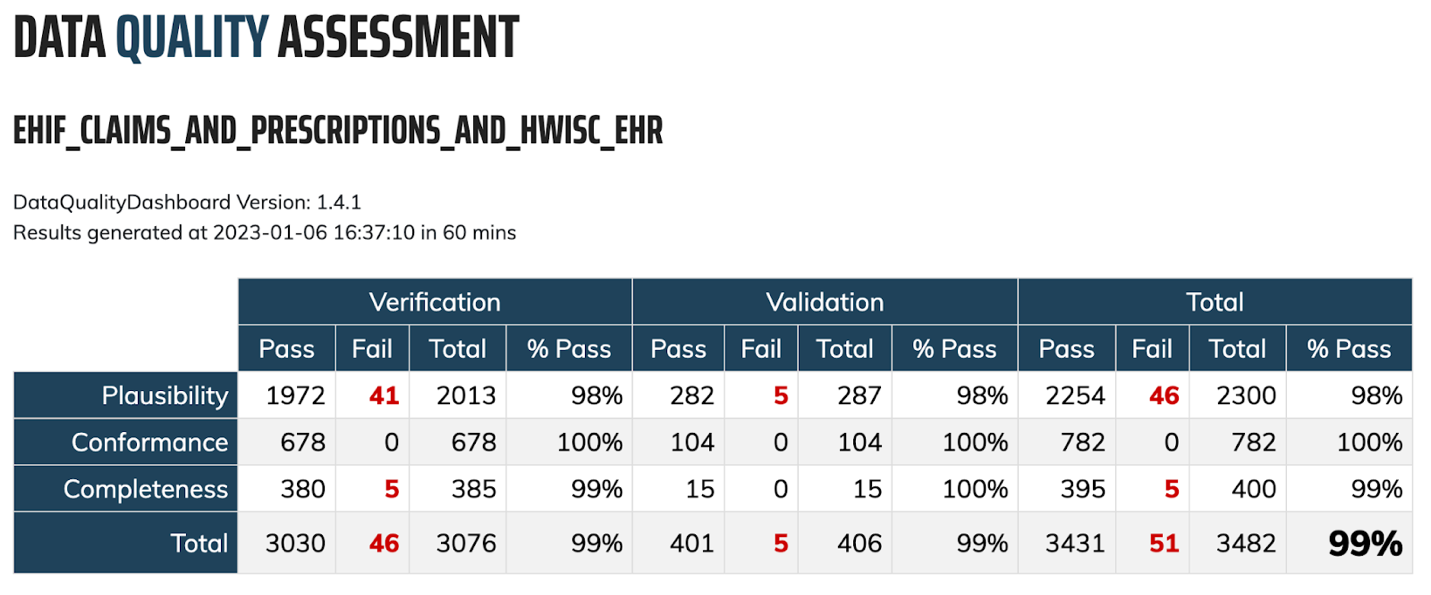


**Supplementary Table S1.** DataQualityDashboard results of the data transformed to OMOP CDM

Supplement: ooad100_Supplementary_Data [file ooad100_supplementary_data.docx]
